# Primary care practitioners’ experiences managing urinary tract infections: a qualitative evidence synthesis

**DOI:** 10.3399/BJGP.2025.0561

**Published:** 2026-09-22

**Authors:** Henrike Kleuser, Francine Toye, Felix Kannapin, Luca Ghirotto, Sandra Parisi, Alice Serafini, Maria Chiara Bassi, Ildikó Gágyor, Peter K Kurotschka

**Affiliations:** 1 Department of General Practice, University Hospital Würzburg, Würzburg, Germany; 2 Physiotherapy Research Unit, Oxford University Hospitals NHS Foundation Trust, Oxford, UK; 3 Azienda USL — IRCCS di Reggio Emilia, Reggio Emilia, Italy; 4 Hochschule Fulda, University of Applied Sciences, Fulda, Germany; 5 Department of Biomedical, Metabolic and Neural Sciences, University of Modena and Reggio Emilia, Modena, Italy

**Keywords:** clinical decision making, general practice, primary health care, qualitative research, systematic review, urinary tract infections

## Abstract

**Background:**

Urinary tract infections (UTIs) are common in primary care. Qualitative studies suggest that the way primary care practitioners (PCPs) manage UTIs may be influenced by a complex interplay of factors.

**Aim:**

To explore the views, perceptions, and experiences of PCPs in the management of UTIs.

**Design and setting:**

Qualitative evidence synthesis of studies set in general practice internationally.

**Method:**

Four databases were systematically searched from inception to 28 June 2024, and forward citation searching of included studies was undertaken. Braun and Clarke’s reflexive thematic analysis was used to synthesise findings and the Critical Appraisal Skills Programme (CASP) checklist was applied to assess the quality of studies.

**Results:**

In total, 3956 records were identified and 190 publications reviewed in full. Thirty-two qualitative studies reporting on 677 PCPs were included, generating the following themes: 1) I am confident; 2) … until patient X presents; 3) I am a victim of an overstretched healthcare system; 4) I must weigh up the benefits and impact of antibiotic treatment; and 5) it all comes down to our relationship. Using the CASP checklist, the quality of research was rated as high.

**Conclusion:**

PCPs felt confident in managing UTIs, but uncertainty arose when patient X (children, men, older patients, those with recurrent UTIs) presented and PCPs perceived consultations as more complex. This analysis highlights the persistent and complex clinical challenge of assessing the risks and benefits of prescribing antibiotics. Chronic underfunding, staff shortages, and conflicting provider–patient expectations further complicated care, particularly when patients fall outside the ordinary (patient X).

## How this fits in

Urinary tract infections (UTIs) are common in primary care, yet, to the authors’ knowledge, qualitative evidence on their management has not previously been synthesised into a coherent conceptual model. Drawing on the experiences of 677 primary care practitioners (PCPs), it was found that management becomes more complex in less frequently encountered patient groups and atypical presentations. Situations that increase clinical uncertainty and the strategies PCPs use to navigate them were identified. These findings may inform more targeted guidelines, decision support, and training to support appropriate antibiotic use and provider–patient relationships.

## Introduction

Urinary tract infections (UTIs) are among the most common infections, regardless of the setting of care.^1,2^ Between 50% and 60% of adult women experience at least one UTI in their lifetime, and approximately 20%–30% of women with a UTI experience a recurrence.^3,4^ UTIs are less frequent in men: in German health insurance data, for instance, 78.6% of UTI cases occurred in women and 21.4% in men.^5^ Yet, the incidence of UTIs in men increases with age: in patients aged ≥85 years, the same rate as for women has been observed.^6,7^ In children aged ≤6 years, 7% of females and 2% of males have had a UTI.^8^

UTIs are the second most common reason for antibiotic prescription but have become increasingly challenging to treat because of rising rates of antimicrobial resistance.^9–11^ Although a short, 3-day course of antibiotics is recommended for uncomplicated lower UTIs,^12^ the concern for wellbeing and consideration of individual circumstances as well as bacterial resistance also have an impact on treatment decisions.^13,14^

Many patients with symptoms of a UTI consult a primary care practitioner (PCP), usually their GP.^15^ Qualitative methods offer valuable insights into understanding how PCPs diagnose and treat UTIs, including their decision making.^16–18^ Prior studies suggest that multiple factors can be at play; healthcare-system pressures, such as time constraints, personnel shortages, limited follow-up capacity, and unpredictable consultations, can shape UTI management.^16,19,20^ Patient–clinician interactions, including patient preferences, antibiotic prescribing habits, or PCPs’ familiarity with specific medications, also appear to influence treatment decisions.^21,22^ The wide variation in symptom presentation and disease severity may further complicate care, particularly in patients with known risk factors (such as catheter use), or in groups less frequently seen in general practice, such as children or men.^1,13^ In these situations, recognising symptoms and selecting diagnostic and treatment strategies may be especially demanding.^23,24^

Given this complexity, the aim of the study was to systematically identify qualitative studies exploring PCPs’ views, perceptions, and experiences of managing UTIs in general practice, and to conduct a qualitative evidence synthesis (QES) generating themes that capture shared experiences across professional roles and health systems.^25,26^

## Method

The study followed the Enhancing Transparency in Reporting the Synthesis of Qualitative Research (ENTREQ) guidelines to ensure transparency and quality in reporting.^27^ As QES consists of previously published studies, ethical approval was not sought. The study was prospectively registered at PROSPERO on 4 February 2022 (reference: CRD42022301250).

### Selecting relevant studies

A comprehensive search was performed using multiple search terms for the keywords ‘urinary tract infection’ and ‘qualitative research’ (see Box 1 for the STARLITE report,^28^ and Supplementary Table S1 for the full search strategy). An information specialist (the seventh author) searched Medline, Embase, PsycINFO, and CINAHL from inception to 6 October 2022 (last update: 28 June 2024). A forward citation search was performed of all included studies on 28 April 2025, using the citationchaser R Shiny app.^29^

Studies were eligible if they used a qualitative or mixed-methods design to explore PCPs’ experiences treating patients with UTIs. PCPs could include GPs, nurses, practice managers, physician assistants, or other staff (such as receptionists). After deduplication using EndNote, the ninth author and another researcher (the first or third authors) independently reviewed titles and abstracts in Rayyan.^30^ The same reviewers independently screened the full texts. For studies covering multiple topics or settings, only those with clearly distinguishable narratives were included. Inclusion was limited to studies in English, German, Spanish, or Italian, reflecting the researchers’ native languages. Disagreements were resolved through discussion with a third investigator (the fourth author).

**Box 1.** STARLITE report^28^ — search strategy

Sampling strategy	Comprehensive
**T** ype of studies	Qualitative research, fully reported; mixed methods where qualitative findings can be deciphered
**A** pproaches	Electronic databases and forward citation screening of all included studies on 28 April 2025
**R** ange of years	Inception to 6 October 2022, update 28 June 2024
**L** imits	English, German, Spanish, or Italian, reflecting the researchers’ mother tongues
**I** nclusion and exclusions	Inclusion: study participants: primary care practitioners; context: general practice; and study population: UTI regardless of age/gender Exclusion: secondary care and stakeholders with conflicting (economic or financial) interests about producing/implementing medical devices for UTI treatment and care
**T** erms used (Medline)^a^	**Qualitative methods — thesaurus (MeSH) terms** “Focus Groups”[MeSH] OR “Anthropology, Cultural”[MeSH] OR “Qualitative Research”[MeSH] OR “Nursing Methodology Research”[MeSH] OR “Interviews as Topic”[MeSH] OR “Grounded Theory”[MeSH] OR “Empirical Research”[MeSH] OR “Behavior Observation Techniques”[MeSH] **Qualitative methods — free text** qualitat*[Title/Abstract] OR narrat*[Title/Abstract] OR “mixed-method*”[Title/Abstract] OR “mixed method*”[Title/Abstract] OR “qualitative design”[Title/Abstract] OR “qualitative survey”[Title/Abstract] OR “qualitative questionnaire”[Title/Abstract] OR abductive analys*[Title/Abstract] OR “depth interview*”[Title/Abstract] OR “in-depth interview*”[Title/Abstract] OR “face interview*”[Title/Abstract] OR “interpretative phenomenological analysis”[Title/Abstract] OR “appreciative inquiry”[Title/Abstract] OR postmodern*[Title/Abstract] OR “post modern*”[Title/Abstract] OR “post-modern*”[Title/Abstract] OR poststructural*[Title/Abstract] OR “post structural*”[Title/Abstract] OR “post-structural*”[Title/Abstract] OR “social construct*”[Title/Abstract] OR humanistic[Title/Abstract] OR existential[Title/Abstract] OR experiential[Title/Abstract] OR feminis*[Title/Abstract] OR “semi-structured interview*”[Title/Abstract] OR “semistructured interview*”[Title/Abstract] OR “unstructured interview*”[Title/Abstract] OR “structured interview*”[Title/Abstract] OR “co operative inquir*”[Title/Abstract] OR “co-operative inquir*”[Title/Abstract] OR “digital record”[Title/Abstract] OR audiorecord*[Title/Abstract] OR “action research”[Title/Abstract] OR participant* observ*[Title/Abstract] OR “field note*”[Title/Abstract] OR “field record*”[Title/Abstract] OR fieldnote*[Title/Abstract] OR “theoretical sampl*”[Title/Abstract] OR “purposive sampl*”[Title/Abstract] OR “lived experience*”[Title/Abstract] OR “life experience*”[Title/Abstract] OR “narrative analys*”[Title/Abstract] OR “discourse analys*”[Title/Abstract] OR “conversation analys*”[Title/Abstract] OR (grounded[Title/Abstract] AND (theor*[Title/Abstract] OR stud*[Title/Abstract] OR research[Title/Abstract] OR analys*[Title/Abstract])) OR “constant compar*”[Title/Abstract] OR ((hermeneutic*[Title/Abstract] OR heidegger*[Title/Abstract] OR husserl*[Title/Abstract] OR colaizzi*[Title/Abstract] OR giorgi*[Title/Abstract] OR glaser[Title/Abstract] OR strauss[Title/Abstract] OR (van[Title/Abstract] AND kaam*[Title/Abstract]) OR (van[Title/Abstract] AND manen[Title/Abstract])) AND (ricoeur[Title/Abstract] OR spiegelberg*[Title/Abstract] OR merleau[Title/Abstract])) **Condition terms — thesaurus (MeSH) terms** “Urinary Tract Infections”[MeSH] OR “Cystitis”[MeSH] OR “Pyelonephritis”[MeSH] OR “Pyuria”[MeSH] **Condition terms — free text** Cystitis[Title/Abstract] OR Pyeloneph*[Title/Abstract] OR Pyuria[Title/Abstract] OR “urinary tract infection*”[Title/Abstract] OR UTI[Title/Abstract]
**E** lectronic sources	Medline, Embase, PsycINFO, and CINAHL

a Terms used in other databases are provided in Supplementary Information S1. UTI = urinary tract infection.

### Assessing the study quality

This study used the Critical Appraisal Skills Programme (CASP) to assess the quality of the included studies.^31^ Guided by the 10 CASP questions, the first and ninth authors independently appraised all included studies and resolved any disagreements through discussion.

### Extracting the data

The result section of each study was uploaded to a Microsoft Word document, used for line-by-line coding. The dataset was then transferred to NVivo (version 15.0.0) for further analysis.

### Analysing the data

In the current study the authors considered several synthesis approaches, including meta-ethnography. The latter was deemed less suitable because the aim was not to generate a new interpretive theory but to identify and explore patterns across a diverse dataset of PCPs’ experiences. Reflexive thematic analysis (RTA) was selected as it offers flexibility for conceptual development and allows engagement with both semantic and latent meanings, which aligned with the study’s interpretive paradigm and multidisciplinary team approach. RTA also made it possible to incorporate nuances across studies without imposing a rigid theoretical framework, thereby enhancing reflexivity and depth of interpretation.^32^ RTA involves six stages: familiarisation with the dataset, coding, generating initial themes, developing and reviewing themes, refining and defining themes, and writing up the report.^32^

After familiarisation with the data by reading the whole dataset multiple times and making notes, two reviewers (the first author and either the second, fifth, sixth, or ninth author) coded the dataset line by line. The aim was not to agree on specific codes, but to incorporate additional nuances captured by the second researcher and enrich the analysis. The first author then iteratively clustered codes into candidate themes using NVivo. All authors collaboratively reviewed and refined themes, and produced a final report.

The study’s multidisciplinary team included nine researchers based in Germany, the UK, and Italy: four primary care physicians (the second, the sixth, and the two senior authors), two methodologists (the second and fourth authors), one information specialist (the seventh author), and two global health researchers (the first and fifth authors), the first author being a medical student at the time of writing. The authors aimed at incorporating these diverse perspectives, recognising that multiple context-specific subjective realities exist, and that there can be a range of interpretive truths.^33^ Group discussions were pivotal to bring insights from clinical experiences and social sciences together and include as many interpretive layers as possible. As the main analysist, the first author was aware that interpretation was influenced by their global health perspective and therefore kept a research journal to ensure continual self-reflection.^34^

## Results

### Sample and study characteristics

Figure 1 provides an overview of included and excluded studies.^35,36^ A total of 3956 records were identified, with 3345 remaining after removal of duplicates. Of these, 190 reports were reviewed in full. In total, 32 primary studies reporting on the experiences of 677 PCPs were included.

**Figure 1.**
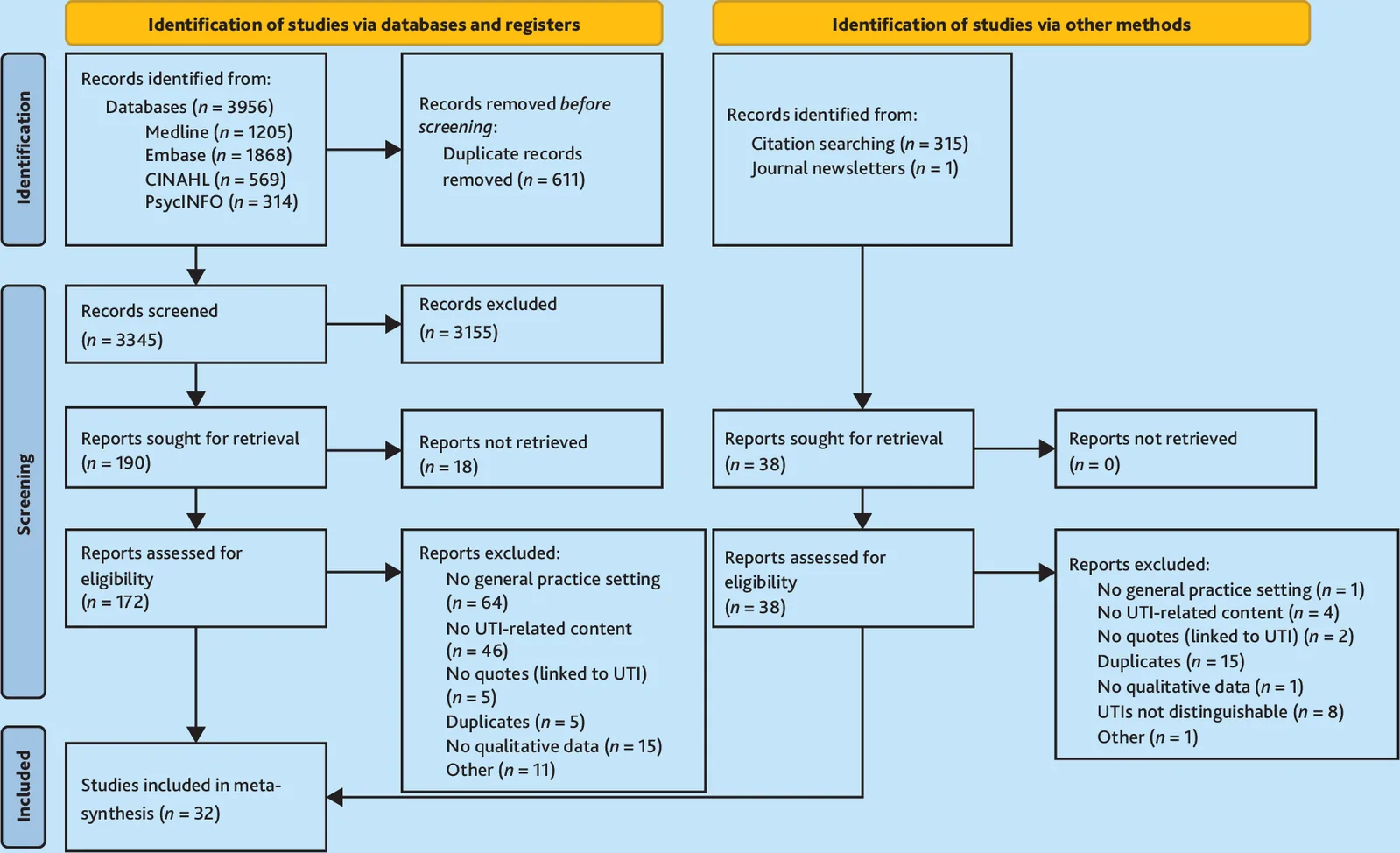
Preferred Reporting Items for Systematic reviews and Meta-Analyses (PRISMA) 2020 flow diagram of included studies.^35,36^ UTI = urinary tract infection.

PCPs included 424 GPs, 73 nurses, 23 physician assistants, 5 healthcare assistants, and 152 other people working in primary care, such as receptionists, administrators, stakeholders, and clinician pharmacists. Specific focuses included childhood UTIs,^37,38^ UTIs in men,^20,39–41^ UTIs in adult women,^14,22,42^ UTIs in older patients,^43–45^ recurrent UTIs,^46,47^ and UTIs in patients aged <65 years.^17^ The remaining studies focused on uncomplicated UTIs,^21,48–54^ or did not specify the type of UTI.^19,55–61^

Ten studies were conducted in the UK,^17,38,39,42,43,45,46,57,58,60^ four in the US,^21,44,47,52^ four in Germany,^14,48,51,61^ three in the Netherlands,^49,53,54^ three in Ireland,^16,19,20^ three in France,^41,50,59^ and two in Sweden.^40,56^ Single studies were conducted in Australia^55^ and Belgium,^37^ and one study included participants from Spain, the Netherlands, and the UK.^22^ Notably, no study was conducted in a low- or middle-income country (LMIC). All studies were published in English, except for one, which was published in German.^51^ See Supplementary Table S1 for characteristics of all included studies.

### Study quality

Using the CASP checklist,^31^ the overall quality of research was rated as high. Most studies stated their aims clearly and used appropriate research designs, as well as recruitment, data collection, and analysis methods. However, only 10 studies explored the relationship between the researcher and participants. A critical reflection on the researcher’s role, perspective, and its influence during the research process was not commonly reported on. See Supplementary Table S2 for the full quality assessment.

### Synthesis of findings

A total of 3064 codes were refined into five final themes:

- I am confident;
- … until patient X presents;
- I am a victim of an overstretched healthcare system;
- I must weigh up the benefits and impact of antibiotic treatment; and
- it all comes down to our relationship.

Each theme is illustrated with exemplary quotations in the main text, with additional supporting quotations provided in Supplementary Box S1.

#### I am confident

PCPs perceived the management of UTIs to be routine, given that patients (predominantly women) frequently presented with UTIs in primary care. PCPs felt confident in their ability to diagnose UTIs, drawing on their clinical experience. There was a sense that the management of UTIs had become a habit. The ease and familiarity with which PCPs managed UTIs was emphasised by this GP:

*‘Well, I think that it is also a bit of a routine* [laughs]*. Kind of, oh it’s cystitis, just treat it, you know* […] *I think it is some sort of convenient behavior, or how can you say it? A reflex-behavior* [laughs]*.’* (GP, the Netherlands)^53^

This confidence stemmed from familiarity with UTI symptoms, and PCPs strongly relied on patient history and their clinical judgement rather than physical examination. Coupled with the established use of antibiotics, managing patients with UTIs initiated a quick and streamlined process, which could be handled remotely:

*‘*… *I don't think I have done a single antibiotic prescription other than UTI without physically laying eyes on someone.’* (GP, Australia)^55^

In some cases, nurses and physician assistants managed uncomplicated UTIs independently, using guidelines to facilitate a streamlined process:

*‘… I deal predominantly in the triage and assessment of acute illnesses and UTIs being probably quite an awful lot of it* … *’* (Nurse prescriber, UK)^17^

In other practices, treatment decisions were made solely by GPs, and the use of guidelines depended largely on the individual provider. One GP felt that there was no need to use guidelines owing to the perceived simplicity of treating UTIs:

*‘There’s very little reason in something as simple as acute cystitis for me to go searching out the guidelines.’* (GP, US)^21^

#### … until patient X presents

When patients presented with symptoms that PCPs could not clearly attribute to a UTI or that indicated a more complicated course, a simple consultation quickly turned into a lengthy process involving accurate diagnosis and treatment. In contrast with routine management, the management of patient X was marked by uncertainty rather than confidence. Patient X includes:

- children;
- men;
- older patients; and
- patients with recurrent UTIs.

Children presented with fever, abdominal pain, vomiting, tenderness, and other symptoms that PCPs could not immediately associate with a UTI. PCPs had to rely on parents’ observations to locate the source of infection, being further challenged by urine collection. For these reasons, PCPs found considering the differential diagnosis of a UTI particularly valuable:

*‘That’s a bit of gut feeling, you can’t really put a finger on that … often high fever, that the child falls asleep on the mom’s lap … no longer drinks anything and has no more pee puddles.’* (GP, Belgium)^37^

*‘Everybody’s pretty aware of signs and symptoms as an adult but with children I think, if I’ve got a bit of tummy pain it could be from anywhere, head to toe really … So I do think they can be difficult from that point of view and yeah I think they do need much more questioning.’* (GP, UK*)* ^38^

As opposed to female patients, male patients often could not place their symptoms when presenting at the practice. PCPs associated these symptoms with sexually transmitted infections and ruled them out first. They also frequently suspected other underlying conditions, prompting more thorough examination and additional diagnostic tests such as C-reactive protein or prostate-specific antigen. Treatment commonly involved a longer duration and an antibiotic perceived to be more potent:

*‘Women seek care with a UTI; men seek care with urinary tract symptoms. But, I guess, it depends on experience, men do not often know what their symptoms stand for. Men have not often experienced it before and do not know anyone who has.’* (GP, Sweden)^40^

Older patients presented with confusion, generalised signs of infection, and multiple ailments unrelated to UTIs. Especially when patients were catheterised, PCPs experienced that diagnostic tests showed an asymptomatic infection, causing uncertainty whether to treat or not. Choice of treatment required careful consideration, as older patients had comorbidities and took multiple medications that increased the risk for interactions:

*‘In fact some of them* [older patients] *may just present as confused without a single solitary abdominal or urinary or clinical examination finding and that’s the absolute truth.’* (GP, Ireland)^20^

Turning to the issue of recurrent UTIs, female patients and PCPs readily recognised the symptoms of recurrent UTIs. However, if the situation remained unchanged, it led to frustration in both groups. In response, PCPs aimed to explore underlying causes. Antibiotic susceptibility testing played an important role although PCPs started empirical antibiotic therapy while awaiting culture results. PCPs considered the use of complementary and alternative medicine as an option:

*‘*[A] *huge number of people who are very prone to urine infections who maybe have been investigated but still get their urine infections and it’s a little bit of a heart sink when they come and see me really.’* (GP, UK*)* ^46^

When managing patient X as a result of a low frequency of such cases in general practice, PCPs felt less experienced, which added to their uncertainty:

*‘I suppose in general practice, you can kind of get into little areas of comfort and I suppose the treatment of male UTIs* […] *wouldn’t be one I’d be hugely comfortable with.’* (GP, Ireland)^20^

The underlying concern of missing a more serious diagnosis, coupled with the perceived complexity of recognising a UTI, influenced PCPs when providing care for patient X. This was heightened by a gut feeling that something was ‘off’ and the fear of persistence or deterioration of symptoms. A GP raised suspicion about UTIs in men:

*‘… you shouldn’t be getting a urinary tract infection … What’s wrong with you? Are you okay? You know, yeah, so it’s almost like backward* [that is, contrary to usual expectations].*’* (GP, Ireland)^20^

When managing patient X, PCPs tried to maintain a sense of safety by running more exhaustive diagnostics and prescribing *‘a stronger, broad-spectrum antibiotic such as ciprofloxacin’* (GP, US),^21^ as well as initiating a longer treatment duration. As opposed to the perceived simplicity of treating uncomplicated UTIs, PCPs supported their management of patient X by using guidelines. However, they were concerned that guidelines were not tailored to individual cases and, therefore, were of limited use for patient X:

*‘No, guidelines can describe some classic cases, they cannot … as a GP you have to take into account … to your judgement and what is actually happening, you cannot always trust in guidelines … Guidelines are just guidelines after all.’* (GP, Sweden)^40^

#### I am a victim of an overstretched healthcare system

PCPs felt that underfunding and staff shortages had a negative impact on UTI consultations. Given the limited time for consultations, PCPs felt pressured to rapidly diagnose UTIs and therefore initiated empirical antibiotic treatment as a quick solution, leaving little room for shared decision making. Consequently, PCPs found themselves more likely to follow patient preferences, as pointed out by this GP:

*‘*… *I think it’s unlikely that a GP can say they’ve never been influenced by a patient to prescribe antibiotics because, at the end of the day, there are certain demands on our time and that does happen, and that sometimes may be for lack of back-up, lack of resources.’* (GP, UK)^43^

As *‘*[i]*t’s all about time’* (GP, UK)^22^ and *‘there are so many other time-consuming tasks to attend to’* (GP, Netherlands),^54^ *‘*[i]*t can be hard to keep up and then some of the little, small tasks get buried’* (resident physician, US),^52^ so that PCPs were in favour of diagnostic tests that produced fast results, were easily available, and easy to use (such as urine dipsticks).

Obtaining a urine sample and diagnosing a UTI did not always fit into a tight schedule, as explained in the case of children:

*‘You can never take ten or five minutes in the hope that the child will urinate during your consultation.’* (GP, Belgium)^37^

Although PCPs emphasised that history taking was essential for diagnosis, they were concerned that in-depth consultations were not remunerated sufficiently. Cost-efficiency was a major concern for PCPs, not only for diagnostic testing but also for treatment:

*‘My personal opinion is that you just have to seek the conversation. That’s the bad thing, after all, what we don’t get paid for anymore, what we don't have time for anymore in General Practice.’* (GP, Germany)^14^

#### I must weigh up the benefits and impact of antibiotic treatment

When selecting a treatment for uncomplicated UTIs, PCPs reported facing decisions about whether to prescribe an antibiotic. PCPs prescribed out of *‘familiarity and comfort’* (GP, US),^21^ fear of symptom deterioration, or because the patient requested it, while acknowledging that this might conflict with accurate and appropriate prescribing:

*‘The more accurate prescribing that there is … probably less resistance there is going to be and we have to balance these global or community issues against the request from the patient sitting in front of you in your surgery.’* (GP, UK)^60^

Although some felt that tailoring prescriptions to patient characteristics might facilitate more effective treatment outcomes and minimise unnecessary medication use, others felt that their decision making was unaffected by concerns about antimicrobial resistance. PCPs thought that the problem was bigger in other countries or that primary care did not contribute to antimicrobial resistance:

*‘I think it’s more relevant in the stationary setting* [that is, the hospital], *because we often don’t have these decisions that require antibiotic stewardship* […] *I think we must set other priorities.’* (GP, Germany)^61^

At the same time, PCPs were particularly wary of the risk of patients developing sepsis or pyelonephritis, resulting in a lower threshold for prescribing antibiotics:

*‘I think certainly any systemic upset, have a low threshold for obviously treating that because you’re worrying about your upper tract infections* … *’* (GP, UK)^43^

#### It all comes down to our relationship

PCPs encountered patients with specific expectations regarding the diagnostic process and treatment of UTIs, feeling that some patients were insistent on identifying the cause of their UTI and on receiving their preferred treatment:

*‘*… *they want to say, so this is happening, and this is happening … they want the diagnosis and of course they want a plan to confirm treatment.’* (GP, Ireland)^20^

At times, this conflicted with PCPs' approach to managing UTIs and challenged the provider–patient relationship. However, this relationship offered an opportunity to discuss treatment options and sustain trust in PCPs:

*‘We live in a free system, and the patient has a free choice* … *you can only avoid* [patients getting antibiotics from other sources] *by building trust* … *’* (GP, Germany)^14^

PCPs were worried about losing patients’ trust if they did not adhere to their expectations. PCPs expressed concern over the dissatisfaction of patients, pointing to their role as an ongoing service provider:

*‘We have a patient population that wants their symptoms to be resolved immediately. Therefore* … *I want to make sure the patient is satisfied and just want to give them something that will resolve symptoms faster.’* (GP, US)^21^

Withholding treatment evoked a sense of guilt among clinicians: even PCPs who were inclined to delay or withhold antibiotics felt pressured to act, wanting to do something for the patient. This guilt was reinforced by recognising that UTI symptoms caused discomfort and distress in patients, potentially resulting in a further consultation:

*‘*[For] *some people, it is also a kind of service orientation, if people have a lot of symptoms and say they cannot cope, then you just cannot wait 24 hours.’* (Nurse, the Netherlands)^22^

However, PCPs developed strategies to manage situations when patient expectations did not align with their own. Shared decision making and patient information were considered the main catalysts for maintaining trust in PCPs and empowering patients:

*‘I think less antibiotic use, that would be the benefit. And of course, the patient’s self-sufficiency* […] *when women see that it* [cystitis] *can resolve spontaneously and they are empowered to handle it themselves, that, of course, saves a bit of time and busyness with us.’* (GP, the Netherlands)^54^

## Discussion

### Summary

This is the first QES, to the authors’ knowledge, that comprehensively explores the views and experiences of primary care professionals when managing acute or recurrent UTIs among patients of all ages and genders, across diverse healthcare systems and countries. The study found that PCPs routinely encountered patients with UTIs and generally felt confident in managing them. However, uncertainty arose when PCPs could not clearly attribute symptoms to a UTI or when patients presented with known risk factors. Consultations for patient X — namely, children, men, older patients, or patients experiencing recurrent UTIs — prompted PCPs to run more exhaustive diagnostics and to initiate a longer, or what they perceived to be ‘stronger’, antibiotic treatment. Yet, chronic underfunding and staff shortages hindered care, especially when patients fell outside ‘routine’. PCPs appeared to weigh the risks and benefits of antibiotic treatment, balancing concerns over rising rates of resistance with the fear of symptom persistence or deterioration when withholding antibiotics. Patient expectations also influenced UTI management, with disagreements or mismatched expectations challenging the provider–patient relationship. See Figure 2 for conceptualisation.

**Figure 2.**
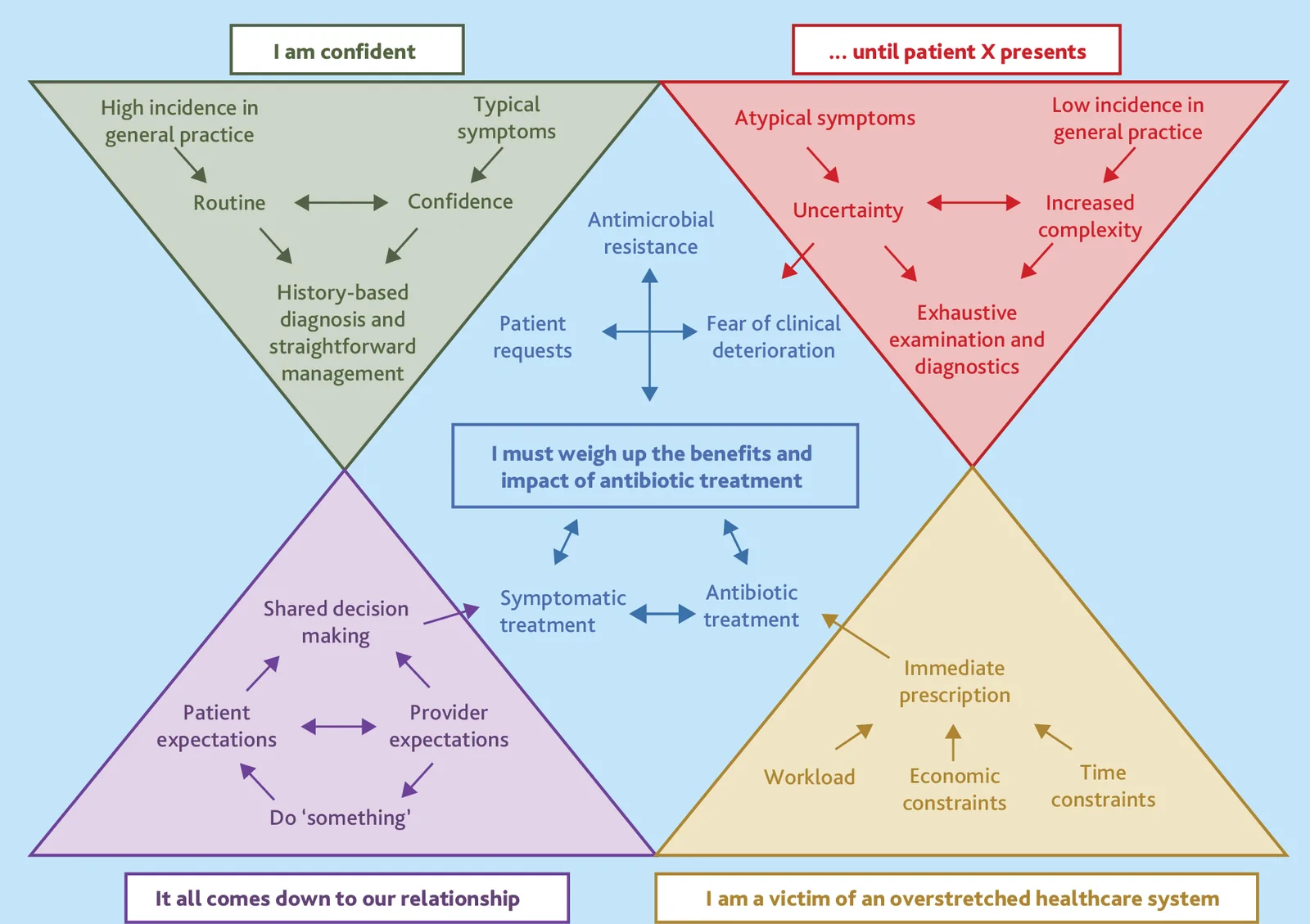
Conceptual model: management of urinary tract infections in general practice.

### Strengths and limitations

The strength of the current study on UTI management lies in providing a holistic understanding of PCPs’ experiences across diverse populations and healthcare systems. A comprehensive strategy was employed searching multiple databases and included a broad range of PCPs and general practice staff from various countries.

Study selection and quality assessment were independently performed by at least two researchers. To ensure trustworthy analysis, the authors collaborated as a multidisciplinary team, making it possible to interpret nuance and meaning from different perspectives.^32,62^

The study has some limitations. First, specific guidance is lacking when applying RTA to QES that calls for *‘the ability to tolerate some degree of uncertainty’*.^32^ Potential limitations of applying RTA to secondary data include the inability to consider non-verbal elements and the lack of insight into how researchers’ presence may have influenced the primary data.^63^ As with all QES approaches, there is a risk of decontextualising findings by removing data from the setting in which they were originally generated.^63,64^ However, this limitation is arguably offset by the opportunity to develop meaning that resonates beyond the original contexts.

Second, the views of all PCPs together were pooled, regardless of their professional background and country. Although this inclusive approach allowed us to capture a wide spectrum of perspectives from those involved in UTI management, it may also be seen as a limitation, as the study does not distinguish between different roles and healthcare systems. Yet, UTI care in general practice is often delivered collaboratively, with overlapping responsibilities. Moreover, the study aimed to explore shared experiences and challenges rather than role-specific practices. Similarly, the authors recognise heterogeneity across healthcare systems and that practice routines may differ by country and system. The aim was, however, to capture overarching themes and shared meaning rather than contrasting experiences and practices.^65^

Third, all included studies were conducted in high-income countries, limiting the transferability of findings to LMICs and reflecting the global imbalance in health research.^65^

### Comparison with existing literature

Overall, PCPs reported confidence in recognising UTI symptoms, usually relying on clinical judgement. Although according to the UK National Institute for Health and Care Excellence guidelines,^12^ along with several other national guidelines,^66–69^ a diagnosis of UTI in women can be made based on key urinary symptoms without the need for further testing if multiple key symptoms are present, this guidance is not universal. For instance, the guideline from the Netherlands recommends dipstick testing in patients with symptomatic cases.^70^ However, the current findings suggest that simple uncomplicated UTIs were generally perceived as straightforward consultations, requiring neither physical examination nor additional diagnostics. Most likely, this confidence was reinforced by the high incidence of UTIs in primary care, accounting for about 1.7% of consultations, with 85% of these involving female patients.^71^

Some patient groups presented less frequently or with ambiguous symptoms (children, males, older adults, and those with recurrent UTIs), making interpretation of symptoms and test results more difficult.^72^ In the current study these are referred to as ‘patient X*’*, a group described in the literature as ‘complicated cases’ or as having ‘complicated features’.^17,22^ Yet, current definitions of what constitutes a complicated UTI vary, being based on host factors, clinical presentation, or a combination of both.^73–75^ In the current study, some PCPs considered UTIs in men as uncomplicated,^40^ whereas others associated them with higher risks of complications and mentioned their low frequency in general practice.^41^ Managing patient X was guided by a shared feeling that something is ‘off’, a gut feeling that Stolper *et al* describe as a pattern that deviates from the expected,^76–78^ evoking a sense of alarm that prompts further enquiry.^79,80^

As a result of rising patient numbers, an ageing population, and stagnant healthcare funding, PCPs operate under growing pressure.^81^ In the UK, for instance, spending on genitourinary medicine services declined by 3.5% between 2014 and 2016, despite an increase of a third in patient attendances from 2011 to 2015.^82^ Cuts to preventive and outreach services further burdened general practice, with PCPs expected to partly absorb hospital-level care.^81,82^

Evidence from the UK suggests that rising uptake of private services increasingly places private health care under pressure.^83^ Although PCPs working in private practices in the US report slightly better working conditions,^84^ mismatches between time allocation and work expectations persist regardless of publicly or privately funded healthcare systems.^84,85^ Working under *‘fictive schedules and* [an] *unbearable load’* ^86^ with demand increasingly exceeding capacity^87^ is mirrored in ‘I am a victim of an overstretched healthcare system’*.*

Although antibiotics remain the most used treatment for UTIs, non-antibiotic strategies could be successful in reducing antimicrobial resistance.^88,89^ Studies show that patients tend to be concerned about antimicrobial resistance,^90–92^ often using antibiotics as a last resort,^91–93^ and even expressing anger that alternative treatments are not the subject of discussions.^94^ ‘I must weigh up the benefits and impact of antibiotic treatment’ reflects the need to ‘do something’ for the patient although contributing to the overuse of antibiotics.^95^

Previous studies showed that treatment decisions can create potential tension in the provider–patient relationship, with some patients perceiving PCPs as unempathetic, dismissive, or indifferent to their symptoms.^92,96^ Altiner *et al* showed that enhancing the provider–patient relationship minimises antibiotic use for acute cough in primary care.^97^ By employing consulting strategies that make patient expectations explicit, unwanted antibiotics may be reduced while maintaining a good relationship,^98^ an aspect covered in the theme ‘It all comes down to our relationship’*.*

### Implications for research and practice

Although the current study focused on the management of patient X — children, men, older patients, and those with recurrent UTIs — the authors acknowledge that other groups, such as pregnant women, may present with additional complexities that have not been covered in this study. Further qualitative studies are needed to identify which other groups may fall under patient X, what factors contribute to clinical uncertainty in UTI management, and how these complexities are perceived across different health settings (such as secondary care) and specialties (such as urology, paediatrics, and gynaecology).

Moreover, investigation of professional groups within primary care teams would help to identify role-specific barriers and tailor targeted interventions accordingly. In addition, joint exploration of patient and provider views, for example, in focus groups, could shed light on shared or contrasting perspectives on antimicrobial resistance. Finally, research from LMICs is needed to better understand how social and cultural contexts influence UTI diagnosis and treatment.

Given the current findings, needs-oriented support for practice teams, such as access to evidence-based decision-support algorithms, may facilitate the identification of atypical presentations and the early recognition of deterioration, particularly when uncertainty arises. The doctor–patient relationship is central to UTI management and depends on sufficient time and resources for discussion and shared decision making, supported by clear communication of the generally self-limiting course of the disease. To address patient expectations, delayed prescription can be used as a strategy to mitigate antibiotic use while also serving as a safety-netting approach for PCPs.

## Data

All data underlying this review are published and publicly available.
